# Alpha-hemolysin promotes internalization of *Staphylococcus aureus* into human lung epithelial cells via caveolin-1- and cholesterol-rich lipid rafts

**DOI:** 10.1007/s00018-024-05472-0

**Published:** 2024-10-16

**Authors:** Oliver Goldmann, Julia C. Lang, Manfred Rohde, Tobias May, Gabriella Molinari, Eva Medina

**Affiliations:** 1 Infection Immunology Research Group, Helmholtz Centre for Infection Research, 38124 Braunschweig, Germany; 2 Central Facility for Microscopy, Helmholtz Centre for Infection Research, 38124 Braunschweig, Germany; 3 InSCREENeX GmbH, Inhoffenstrasse 7, 38124 Braunschweig, Germany; 4 Present Address: AIMES-Center for the Advancement of Integrated Medical and Engineering Sciences, Department of Neuroscience, Karolinska Institutet and KTH Royal Institute of Technology, Stockholm, 171 77 Sweden

**Keywords:** *Staphylococcus aureus*, Human lung epithelial cells, Lipid rafts, Bacterial internalization, Alpha-hemolysin, Caveolin-1

## Abstract

*Staphylococcus aureus* is a pathogen associated with severe respiratory infections. The ability of *S. aureus* to internalize into lung epithelial cells complicates the treatment of respiratory infections caused by this bacterium. In the intracellular environment, *S. aureus* can avoid elimination by the immune system and the action of circulating antibiotics. Consequently, interfering with *S. aureus* internalization may represent a promising adjunctive therapeutic strategy to enhance the efficacy of conventional treatments. Here, we investigated the host-pathogen molecular interactions involved in *S. aureus* internalization into human lung epithelial cells. Lipid raft-mediated endocytosis was identified as the main entry mechanism. Thus, bacterial internalization was significantly reduced after the disruption of lipid rafts with methyl-β-cyclodextrin. Confocal microscopy confirmed the colocalization of *S. aureus* with lipid raft markers such as ganglioside GM1 and caveolin-1. Adhesion of *S. aureus* to α5β1 integrin on lung epithelial cells via fibronectin-binding proteins (FnBPs) was a prerequisite for bacterial internalization. A mutant *S. aureus* strain deficient in the expression of alpha-hemolysin (Hla) was significantly impaired in its capacity to enter lung epithelial cells despite retaining its capacity to adhere. This suggests a direct involvement of Hla in the bacterial internalization process. Among the receptors for Hla located in lipid rafts, caveolin-1 was essential for *S. aureus* internalization, whereas ADAM10 was dispensable for this process. In conclusion, this study supports a significant role of lipid rafts in *S. aureus* internalization into human lung epithelial cells and highlights the interaction between bacterial Hla and host caveolin-1 as crucial for the internalization process.

**Supplementary Information:**

The online version contains supplementary material available at 10.1007/s00018-024-05472-0.

## Introduction

*Staphylococcus aureus* is an important cause of lung infections and a leading cause of ventilator-associated pneumonia in intensive care units [1, 2]. The respiratory epithelium spans both the upper and lower airways and extends into the lung alveoli. This broad coverage positions lung epithelial cells as the first point of contact with respiratory pathogens, including *S. aureus.* In this regard, several studies have confirmed the ability of *S. aureus* to internalize within lung epithelial cells [3–7]. This internalization strategy potentially shields *S. aureus* from the attack of professional phagocytic cells, which would otherwise aim to eliminate the extracellular bacteria [3–7]. In addition, internalized *S. aureus* can evade the killing effects of circulating antibiotics, as these antimicrobial agents may have limited access to the intracellular compartment [3–7]. Suboptimal intracellular antibiotic concentrations can lead to reduced efficacy in eliminating intracellular *S. aureus* and promote pathogen persistence. The suboptimal antibiotic concentrations can also create an environment that favors the selection and survival of antibiotic-resistant strains. Unlike professional phagocytic cells such as macrophages and neutrophils, epithelial cells are generally considered to be non-phagocytic. The traditional role of respiratory epithelial cells is to form protective barriers and to orchestrate the immune response to pathogens rather than actively engulf and digest them [8]. Therefore, the process of bacterial internalization into respiratory epithelial cells is often self-promoted by the pathogen [9]. Despite the recognized ability of *S. aureus* to internalize into lung epithelial cells, the precise mechanism of bacterial entry remains an active area of research. Identifying and targeting the molecular events involved in bacterial internalization may provide potential avenues for therapeutic intervention.

The internalization of pathogens into eukaryotic cells can occur through several cellular processes, including clathrin-mediated and clathrin-independent endocytosis [10]. Clathrin-mediated endocytosis is a fundamental cellular process used by eukaryotic cells for the internalization and recycling of surface receptors [11]. The initiation of clathrin-mediated endocytosis is characterized by the formation of coated pits, which are enriched with clathrin and other associated proteins, at the cell surface [11]. The coated pits invaginate from the cell surface, resulting in the formation of coated vesicles containing cargo molecules inside of the cells [11]. The internalized cargo, which may include receptors, can be recycled back to the cell surface or targeted to specific cellular compartments for degradation. Although the primary role of the clathrin machinery is to internalize cell surface receptors to regulate their abundance on the cell surface [12], it is often manipulated by pathogens to gain access into host cells [13, 14]. A possible role for clathrin-dependent endocytosis has been proposed for the entry of *S. aureus* into osteoblasts [15].

Clathrin-independent endocytosis, on the other hand, allows the cell to internalize a wide variety of cargo, including extracellular ligands, receptors, and pathogens, without relying on the formation of clathrin-coated vesicles [16]. The majority of clathrin-independent endocytic pathways take place in lipid rafts, which are specialized membrane microdomains that are enriched with cholesterol, glycophospholipids, and lipid-anchored membrane proteins [17, 18]. Many pathogens exploit lipid rafts for entry into host cells [19–25]. Pathogens that enter host cells via lipid rafts follow a different pathway than clathrin-dependent endocytosis [26]. Whereas the endocytic vesicles formed by clathrin-dependent endocytosis are typically targeted to intracellular compartments that fuse with lysosomes, pathogens entering host cells via lipid rafts are targeted to intracellular compartments that do not fuse with lysosomes [26]. Therefore, pathogens may strategically use lipid rafts as entry points to evade intracellular degradation and survive within the host cell. In this regard, internalization of *S. aureus* in fibroblasts has been reported to be mediated by sphingolipid- and cholesterol-rich lipid rafts [27].

The interactions between *S. aureus* and host cells are highly complex and can vary depending on the specific cell type involved. Different cell types express unique sets of receptors and signaling pathways that can influence the dynamics of infection. The primary objective of the present study was to investigate the mechanisms by which *S. aureus* internalizes into human lung epithelial cells. Our results provide compelling evidence for an important role of lipid rafts in bacterial internalization. The key finding of this study is that alpha-hemolysin (Hla) released by *S. aureus* specifically interacts with caveolin-1 within lipid rafts in the host cell membrane and that this interaction is critical for bacterial internalization. Hla is a major pore-forming toxin secreted by *S. aureus* and has been reported to interact with lipid rafts in host cells [28–30]. Caveolin-1 is one of the principal structural proteins associated with lipid rafts and acts as a scaffold or organizing platform for the assembly of these membrane microdomains [31]. The results of our study suggest a model in which Hla produced by *S. aureus* interacts with caveolin-1 on lung epithelial cells, leading to lipid raft formation in the host cell membrane and bacterial internalization.

## Materials and methods

### Bacterial strains and growth conditions

The strains of *S. aureus* used in this study were *S. aureus* strain SH1000 [32], the Hla-deficient (*hla*) mutant strain [33], *S. aureus* 8325-4 and its fibronectin-binding protein A-deficient (*FnBPA*) derivative [34], *S. aureus* USA300 [35] and the clinical isolates SA102, SA103, SA112, SA138 and SA302 obtained from our strain collection. *S. aureus* strains were grown to mid-log phase in Brain-Heart Infusion (BHI) medium (Roth) at 37 °C with shaking (120 rpm). Bacteria were collected by centrifugation, washed with sterile PBS, and diluted to the required concentration.

For carboxyfluorescein labeling of *S. aureus*, a bacterial suspension (5 × 10^8^/ml) was incubated with 200 µg/ml of carboxyfluorescein (Sigma-Aldrich) in PBS at 4 °C for 30 min in the dark. Labeled bacteria were washed three times before use.

### Culture of human lung epithelial cells

The human adenocarcinomic alveolar basal epithelial cells A549 (ATCC CCL-185) were cultured in Dulbecco’s Modified Eagle Medium (DMEM; GIBCO) supplemented with 10% FCS and the immortalized human bronchial basal epithelial cells CI-huBroBECs (InSCREENeX; #INS-CI-1025) were cultured in huBroBEC medium (InSCREENeX; #INS-ME-1033). Cells were incubated in 5% CO_2_ in an air-humidified incubator at 37 °C. The media was changed every 2 days, and cells were passaged when they were approximately 80% confluent.

### Antibodies and reagents

The following antibodies and reagents were used in this study: mouse anti-caveolin-1 (BD Bioscience); mouse anti-lysosomal-associated membrane protein (LAMP-1) antibody (BD Bioscience); cross-adsorbed secondary antibodies (goat anti-rabbit or goat anti-mouse IgG) conjugated with Alexa Fluor 488 or Alexa Fluor 568 (Thermo Fisher Scientific); Alexa Fluor 488-conjugated Phalloidin (Thermo Fisher Scientific); BODIPY 493/503 (Thermo Fisher Scientific); and Alexa Fluor 594 cholera toxin subunit B (CT-B) conjugate (Thermo Fisher Scientific). The rabbit polyclonal anti-*S. aureus* antibodies used in this study were custom-made.

### Infection assay for quantification of viable intracellular *S. aureus*

Human lung epithelial cells were seeded in 24-well tissue culture plates (Nunc) (5 × 10^5^ cells/well) and infected with *S. aureus* at a multiplicity of infection (MOI) of five bacteria per cell (10^6^ bacteria/well). The multiplicity of infection of 5 bacteria per eukaryotic cell was identified in preliminary experiments as the optimal MOI that balances sufficient infection of lung epithelial cells and host response to initial infection with minimal cytotoxic effects for the host cells. Plates were centrifuged at 800 x *g* for 1 min to synchronize infection. After 2 h of infection, lysostaphin (2 µg/ml) (Sigma-Aldrich) was added and cells were incubated for 10 min to remove non-internalized extracellular bacteria. Cells were then washed twice with sterile PBS and lysed at the indicated times by incubation with 0.1% Triton X-100 in double-distilled H_2_O for 5 min. The number of viable bacteria was determined by plating serial dilutions on blood agar plates. The limit of detection was less than 50 CFU/ml.

In some experiments, lung epithelial cells were incubated with either 10 µM or 30 µM Pitstop 2 (Sigma-Aldrich) to inhibit clathrin-mediated endocytosis [36], 100 µM RGD peptide (GLY-ARG-GLY-ASP-SER-PRO) (Sigma Aldrich) to block the binding of fibronectin to host integrin α5β1 [37] or with reverse sequence RGD peptide as a negative control, 10 mM of cholesterol-depleting agent methyl-β-cyclodextrin (MβDC; Sigma-Aldrich), 50 nM or 100 nM of the selective disruptor of caveolin-1 oligomers WL47 [38], and 1–10 µg of a caveolin-1 blocking peptide (Bio-Connect). Cells were preincubated with these compounds for 1 h before and during infection at the indicated concentrations while control cells were incubated with vehicle only.

### Infection assay for fluorescence microscopy

Human lung epithelial cells were seeded on glass coverslips in 24-well tissue culture plates (Nunc) in DMEM medium supplemented with 10% FCS to obtain sub-confluent cell monolayers. The cells were infected with *S. aureus* at a MOI of five bacteria per cell. At the indicated time points of infection, the coverslips were washed twice with PBS and fixed with 3% paraformaldehyde in PBS for 30 min at RT. Prior to fluorescence labeling, the coverslips were first washed with 10 mM glycine in PBS to quench free aldehyde groups, followed by two washes with PBS and were then blocked in 10% FCS-PBS for 45 min. Infected cells on coverslips were stained as described below. Stained coverslips were washed with PBS and mounted on glass microscope slides using Prolong Gold antifade mounting medium (Thermo Fisher Scientific) (with or without DAPI), sealed with nail polish and stored at 4 °C.

Samples were analyzed either using either a Zeiss Imager A2 (Zeiss, Germany) with a 63x/1.25 oil Ph3 (Plan Neofluar) or a 100x/1.25 oil Ph3 (Plan Neofluar) objective and an Axiocam MRm camera or under a Leica TCS SP5 laser scanning confocal upright microscope (Leica Microsystems) with an HC PL APO 63×/1.40 oil immersion objective and two lasers, diode (405) and argon (488 nm), and the LAS AF software. The image analysis was performed using Zeiss Zen 2011 blue software for images taken with Zeiss Imager A2 or with Fiji/ImageJ [39] for images taken with Leica TCS SP5 laser scanning confocal.

### Double immunofluorescence staining for detection of extracellular/intracellular *S. aureus* 

After the blocking step described above, the coverslips were incubated with a 1:100 dilution of rabbit *S. aureus* antiserum (primary antibody) for 1 h without washing. After three washes with PBS, samples were incubated with secondary antibody Alexa Fluor 488-conjugated goat anti-rabbit (1:150) for 45 min to stain extracellular bacteria. The coverslips were then washed three times, permeabilized with 0.1% Triton X-100 in PBS for 5 min, washed twice and incubated again with the primary antibody for 1 h to stain intracellular *S. aureus*. After three washes, samples were incubated with secondary antibody Alexa Fluor 568-conjugated goat anti-rabbit (1:150). Coverslips were washed and incubated with Alexa Fluor 400 Phalloidin (1:200) for 45 min to visualize F-actin. Coverslips were washed three times before mounting.

For interpretation of the double immunofluorescence images, extracellular bacteria that were initially stained green will appear yellow after the second staining due to the overlap of green and red fluorescence. Intracellular bacteria will appear red because they are stained only with the red fluorescent antibody after permeabilization.

### Immunofluorescence staining for the detection of LAMP-1, caveolin-1, and *S. aureus*

*S. aureus-*infected lung epithelial cells were permeabilized with 0.1% Triton X-100 in PBS for 5 min and washed twice with PBS before the blocking step. Coverslips were then incubated with either a mouse anti-LAMP-1 antibody (1:50) or a mouse anti-caveolin-1 antibody (1:250) for 1 h, washed twice, and incubated with Alexa 488-conjugated goat anti-mouse (1:150) secondary antibody. After two washes and incubation with blocking buffer for 5 min, the coverslips were further incubated with rabbit *S. aureus* antiserum (1:100) for 1 h without washing. The samples were washed twice and incubated with Alexa 568-conjugated goat anti-rabbit secondary antibody (1:150). Coverslips were washed three times before mounting.

### Fluorescence labeling of lipids with BODIPY 493/503

The fluorescence staining of the lipid droplets was performed with BODIPY 493/503 (Thermo Fisher Scientific) in accordance with the manufacture’s recommendations.

### Fluorescent labeling of ganglioside GM1 using cholera toxin B subunit (CTB)

*S. aureus-*infected lung epithelial cells were incubated with CTB (1:50) for 20 min, washed twice, blocked for 5 min and further incubated with rabbit anti-*S. aureus* antibody (1:100) for 30 min without washing. After incubation, the samples were washed twice and incubated with Alexa 488-conjugated goat anti-rabbit (1:150) secondary antibody. Coverslips were washed three times before mounting. No permeabilization of the cell membranes was performed during this labeling. Thus, only extracellular bacteria were labeled.

### Field emission scanning electron microscopy (FESEM)

*S. aureus-*infected and uninfected control lung epithelial cells were fixed with 2% glutaraldehyde and 5% paraformaldehyde in 0.1 M EM-HEPES. After fixation, cells were washed twice with TE buffer (20 mM Tris, 1 mM EDTA [pH 6.9]) and dehydrated with a graded series of acetone (10, 30, 50, 70, 90, 100%) for 15 min on ice. Samples in the 100% acetone were allowed to reach room temperature before being placed in 100% acetone for a second time. Samples were then critical-point dried with liquid CO_2_ (CPD 30, Bal-Tec) and coated with a palladium-gold film by sputter deposition (SCD 500, Bal-Tec). Cells were examined with a field emission scanning electron microscope (Zeiss DSM 982 Gemini) using the Everhart Thornley HESE2 detector and the in-lens SE detector in a 25:75 or 50:50 ratio at an acceleration voltage of 5 kV.

### Transmission electron microscopy (TEM)

*S. aureus-*infected and uninfected control lung epithelial cells were fixed as described above for FESEM. After washing with TE buffer, cells were osmified with 1% aqueous osmium at RT for 1 h. Cells were then dehydrated with a graded series of acetone (10, 30, 50, 70, 90, and 100%) for 30 min at each step. The 70% acetone dehydration step was performed overnight in 2% uranyl acetate. Cells were then infiltrated with an epoxy resin, and ultrathin 70-nm sections were cut with a diamond knife. Sections were counterstained with uranyl acetate and lead citrate and examined with a TEM910 transmission electron microscope (Carl Zeiss) at an acceleration voltage of 80 kV. Images were taken at calibrated magnifications using a line replica and digitally recorded with a slow scan CCD-Camera (ProScan) using ITEM software (Olympus Soft Imaging Solutions). Brightness and contrast were adjusted using Adobe Photoshop CS5.

### Immune field emission scanning electron microscopy (Immune-FESEM)

*S. aureus-*infected and uninfected control lung epithelial cells were fixed with 3% paraformaldehyde in 0.1 M EM-HEPES for 1 h, washed first with 10 mM glycine in PBS to quench free aldehyde groups, and then washed twice with PBS. The cells were first incubated in 10% FCS-PBS for 45 min for blocking and then with 50–100 µg/ml of purified anti-Hla rabbit IgG (S7531, Sigma-Aldrich) for 1 h. After several washes with PBS, the cells were incubated with goat anti-rabbit IgG-gold conjugated (particle size 15 nm) in BBI solution for 30 min. After washing with TE buffer, cells were fixed in 1% glutaraldehyde in TE and dehydrated in graded series of acetone, as described for TEM. Cells were then coated with a thin carbon layer using a Bal-Tec MED 020 (Liechtenstein), which allows the detection of colloidal gold particles on bacterial and cellular surfaces without charging problems, and were finally examined in a field emission scanning electron microscope (Zeiss DSM 982 Gemini) using the Everhart Thornley HESE2 detector or the In-lens SE detector at an acceleration voltage of 5 kV.

### Data analysis

Statistical analysis was performed with GraphPad Prism version 9.4.1 software. Differences between two groups were determined using a Student’s t-test. Groups of three or more were analyzed by one-way analysis of variance (ANOVA). 

## Results

### *S. aureus* efficiently internalizes into human lung epithelial cells

**Fig. 1:**
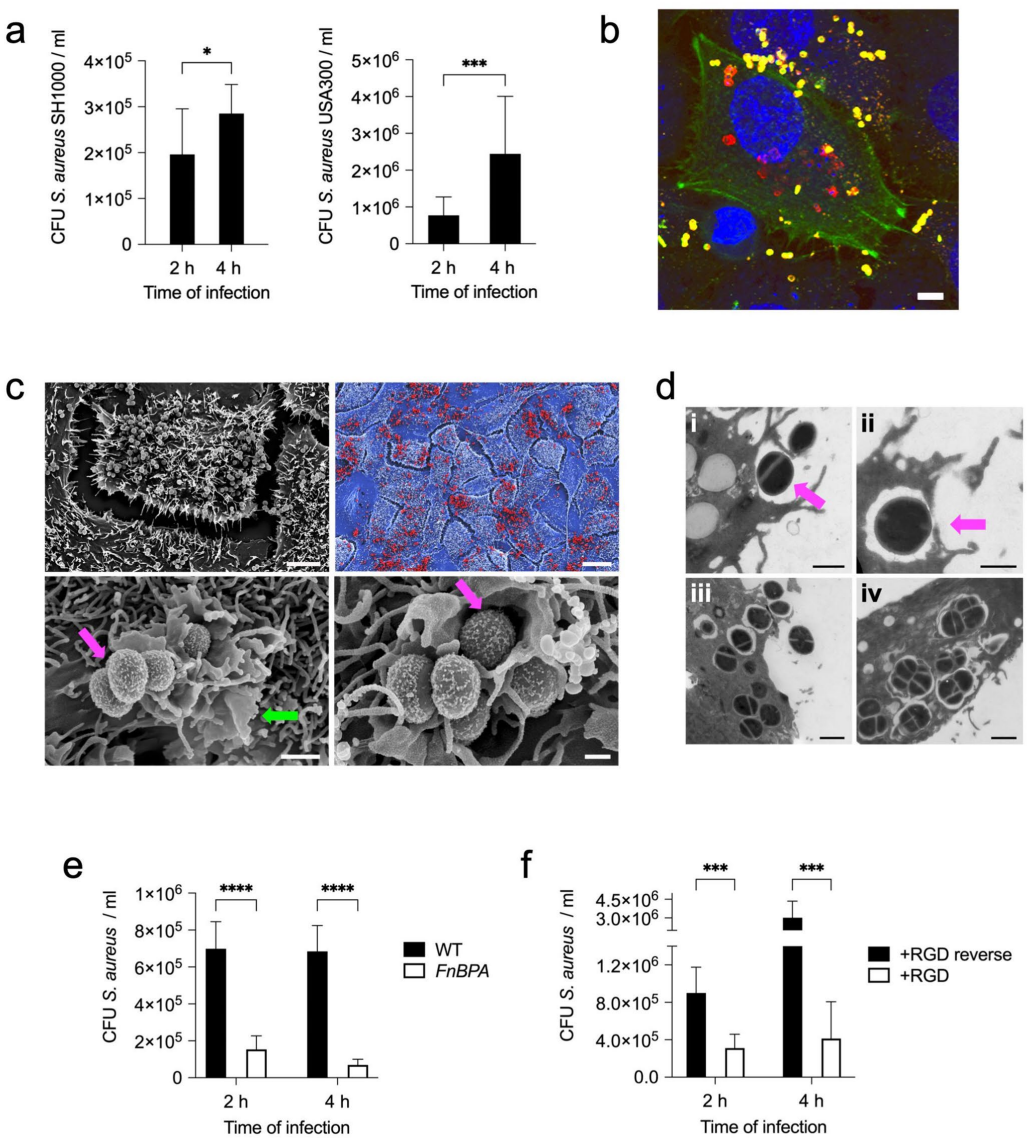
*S. aureus* adheres to and internalizes into human lung epithelial cells. **a** Quantification of viable intracellular *S. aureus* strain SH1000 (left panel) and *S. aureus* strain USA300 (right panel) in A549 cells at 2 h and 4 h of infection. **b** Double immunofluorescence staining image of A549 cells infected with *S. aureus* SH1000 at 4 h of infection showing the intracellular bacteria in red, extracellular bacteria in yellow, actin in green and DNA in the nucleus is stained in blue. The scale bar represents 5 μm. **c** Field emission scanning electron microscopy (FESEM) photographs showing A549 cells infected with *S. aureus* SH1000 (4 h) at different magnifications. The photograph in the upper right panel is a digitally colorized version showing a detail of the image in the left panel with *S. aureus* bacteria colored in red and A549 cells colored in blue. Magenta arrows indicate *S. aureus* bacteria and green arrow indicates membrane ruffles. Scale bars represent 5 μm for the upper left panel, 20 μm for the upper right panel and 0.5 μm for the lower panels. **d** Transmission electron microscopy images showing adhesion (i), engulfment (ii), internalization (iii), and replication (iv) of *S. aureus* SH1000 in A549 cells. Scale bars represent 1 μm for (i) and (iv) and 0.5 μm for (ii) and (iii). **e** Quantification of viable intracellular *S. aureus* strain 8325-4 wild-type (WT) (black bars) and *S. aureus* strain 8325-4 deficient in fibronectin-binding protein A (*FnBPA*) expression (white bars) at 2 h and 4 h of infection. **f** Quantification of viable intracellular *S. aureus* strain SH1000 within A549 cells treated with either RGD peptide (white bars) or with RGD reverse peptide as negative control (black bars). Each bar in **a**, **e** and **f** represents the mean value ± SD of three independent experiments. *, *p* < 0.05, ***, *p* < 0.001, ****, *p* < 0.0001

The capacity of *S. aureus* to internalize into human lung epithelial cells was first evaluated using a lysostaphin-protection assay. In the experimental setup, A549 cells were infected with either *S. aureus* strain SH1000 or *S. aureus* strain USA300. The infection period lasted for 2 h to allow the bacteria to interact and internalize into the host cells. After the 2 h infection period, lysostaphin was added to selectively kill extracellular bacteria while sparing those that had successfully entered the A549 cells. The first set of samples was processed immediately after 10 min of lysostaphin treatment (time point 2 h). This rapid disruption allowed the number of viable intracellular bacteria to be determined at the initial 2 h time point. Another set of samples was incubated for an additional 2 h after lysostaphin treatment (a total of 4 h from the beginning of infection). This additional incubation period provided insight into the fate of intracellular bacteria over a longer period of time. Both *S. aureus* strain SH1000 and *S. aureus* strain USA300 showed efficient internalization into A549 cells after 2 h of infection (Fig. 1a). The number of intracellular viable bacteria increased significantly between 2 h and 4 h of infection (Fig. 1a), indicating that intracellular *S. aureus* was also able to replicate within A549 cells. The ability of *S. aureus* to internalize within human lung epithelial cells was further confirmed using the immortalized human bronchial basal epithelial cells CI-huBroBECs (Supplementary Fig. S1a). The presence of intracellular bacteria in A549 (Fig. 1b, Supplementary Fig. S2a-e) or in CI-huBroBECs cells (Supplementary Fig. S1b) was also demonstrated by confocal microscopy. The confocal microscopy images depicted in these figures show substantial numbers of *S. aureus* internalized within the epithelial cells (red fluorescence) along with extracellular bacteria adhered to the cell surface (yellow fluorescence). Examination of infected A549 by scanning electron microscopy revealed abundant *S. aureus* bacteria attached to the cell surface (Fig. 1c, upper panels). Large membrane raffles were observed on the surface of the A549 cells surrounding the attached bacteria (Fig. 1c, lower panels). The transmission electron microscopy images in Fig. 1d show *S. aureus* at different stages of internalization into A549 cells. The earlier step is the attachment to the host cells and the induction of host cell membrane ruffles surrounding the attached bacteria (i). The resulting membrane ruffles formed in this process then collapse onto the plasma membrane, where they fuse together (ii) and then split to form intracellular vacuoles (iii). Within these vacuoles, *S. aureus* can replicate, leading to the formation of bacterial clusters (iv).

### Adhesion to human lung epithelial cell surface via FnBPs is required for *S. aureus* internalization

Adhesion of *S. aureus* to the surface of lung epithelial cells may be a critical step that precedes bacterial internalization. Fibronectin-biding proteins (FnBPs) are widely recognized as the major adhesins of *S. aureus* [40–44]. FnBPs, expressed on the surface of *S. aureus*, bind to fibronectin, which in turn binds to the α5β1 integrin on the surface of the host cell [42]. Thus, fibronectin serves as a bridge between FnBPs on *S. aureus* and α5β1 integrin on the host cell and facilitates a close interaction between the bacterium and the host cell. FnBPs have been shown to be involved in *S. aureus* adhesion to human respiratory epithelial cells [9, 45]. To determine the relevance of bacterial attachment via FnBPs for the internalization of *S. aureus* into human lung epithelial cells, we evaluated the internalization capacity of a mutant *S. aureus* strain deficient in the expression of fibronectin-binding protein A (*FnBPA*). Adhesion of *S. aureus FnBPA* to the surface of A549 cells was significantly reduced compared to wild-type *S. aureus*, indicating that FnBPA is a major factor in mediating bacterial attachment to these cells (Supplementary Fig. S3). Furthermore, the lack of FnBPA expression also resulted in a significant reduction in the number of internalized bacteria at 2 h and 4 h of infection (Fig. 1e). Thus, the initial adhesion of *S. aureus* to the lung epithelial cell surface via FnBPA appears to be an essential step for subsequent internalization. To further corroborate this requirement, we evaluated the effect of blocking the host α5β1 integrin with an RGD-containing peptide on bacterial internalization. RGD is a peptide sequence that competitively interacts with α5β1 integrin, preventing its engagement with fibronectin [37]. A significant reduction in *S. aureus* internalization into A549 cells was observed after treatment with the RGD peptide (Fig. 1f). These data confirmed that attachment to α5β1 integrin on the surface of A549 cells via FnBPA was a prerequisite for subsequent *S. aureus* internalization.

### *S. aureus* internalizes into human lung epithelial cells via a clathrin-independent pathway

**Fig. 2:**
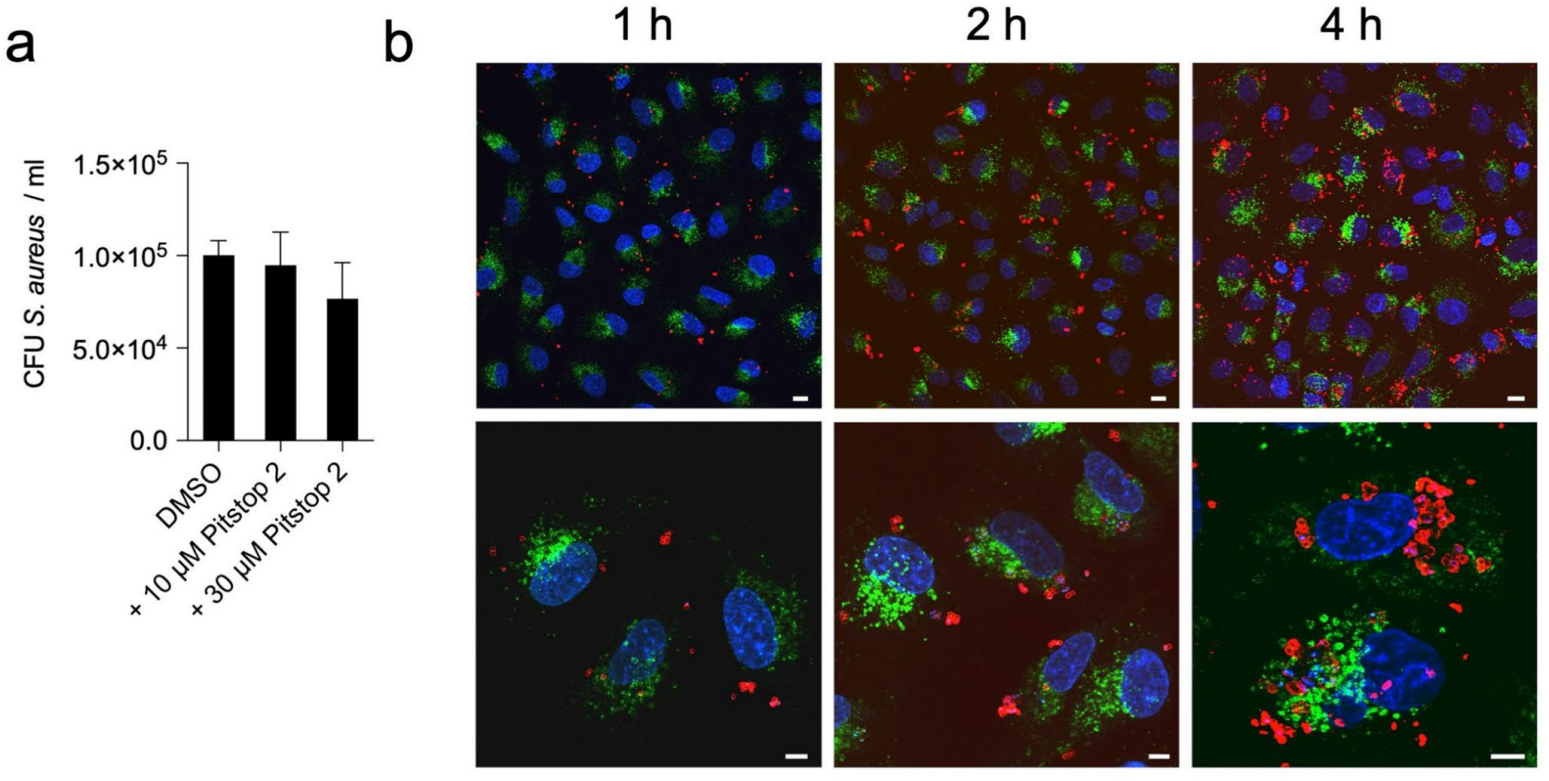
*S. aureus* internalizes into human lung epithelial cells via a clathrin-independent pathway. **a** Quantification of intracellular viable *S. aureus* strain SH1000 at 2 h after internalization into A549 cells treated with either vehicle control DMSO or Pitstop (10 µM and 30 µM). Each bar represents the mean value ± SD of three independent experiments. **b** Confocal microscopy images of *S. aureus* SH1000-infected A549 cells at 1 h (left panels), 2 h (middle panels), and 4 h (right panels) postinfection showing LAMP-1 in green, *S. aureus* in red, and DNA in blue. Scale bars represent 5 μm

After binding to the host cell surface, bacterial internalization can occur via various mechanisms, which can be clathrin-mediated or clathrin-independent [10]. To determine whether *S. aureus* internalization into lung epithelial cells was clathrin-mediated, we monitored the effect of blocking clathrin-mediated endocytosis on bacterial internalization using the selective inhibitor pitstop 2 [36]. The results in Fig. 2a showed that treatment with two different concentrations of pitstop 2 (10 µM and 30 µM) did not affect the internalization of *S. aureus* into A549 cells. Therefore, clathrin-dependent endocytosis may not be the primary pathway used by the bacterium to enter human lung epithelial cells.

While endocytic vesicles generated by clathrin-dependent endocytosis typically fuse with lysosomes, pathogens that enter host cells via clathrin-independent mechanisms such as lipid rafts are targeted to intracellular compartments that do not fuse with lysosomes [26]. The lack of co-localization between intracellular *S. aureus* and LAMP-1, one of the major proteins associated with phago-lysosomes [46], confirmed that the intracellular compartment where *S. aureus* resides within lung epithelial cells does not fuse with lysosomes (Fig. 2b and Supplementary Fig. S4). These observations are in contrast to the reported acquisition of LAMP-1 by *S. aureus* containing vacuoles in immortalized cystic fibrosis tracheal epithelial cells CFT-1 at 1 h after internalization [47]. This discrepancy may be due to different pathways of *S. aureus* internalization between CFT-1 and the lung epithelial cells used in our study, or to a difference in the physiology between the different cell lines.

### Internalization of *S. aureus* into human lung epithelial cells is mediated by lipid rafts

**Fig. 3:**
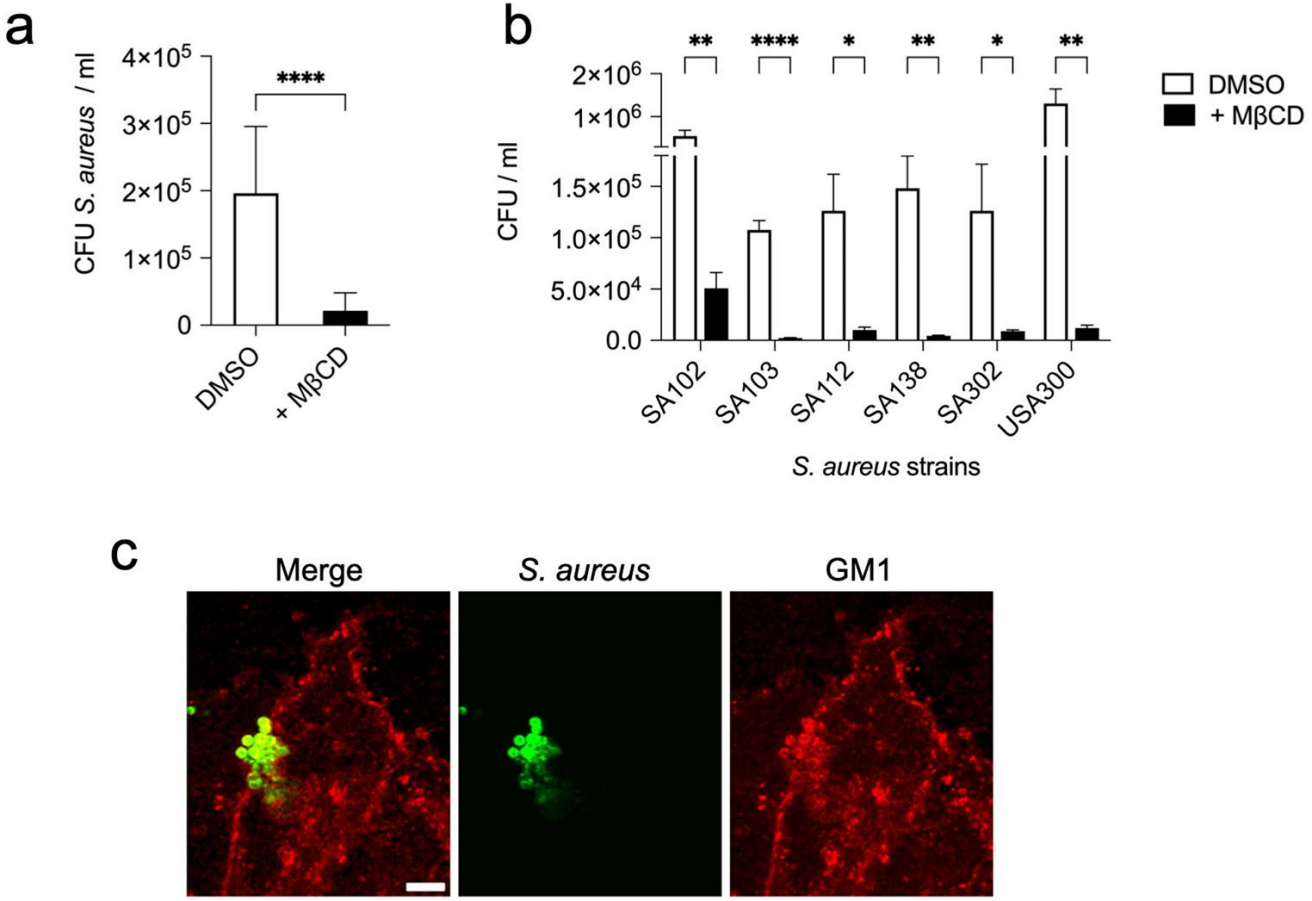
*S. aureus* enters human lung epithelial cells via lipid raft-mediated endocytosis. **a** Quantification of intracellular viable *S. aureus* strain SH1000 at 2 h after internalization within A549 cells treated with either 10 mM MβCD (black bar) or with DMSO vehicle control (white bar). **b** Quantification of viable bacteria within A549 cells infected for 2 h with different *S. aureus* strains and either treated with 10 mM MβCD (black bars) or with DMSO vehicle control (white bars). Each bar in **a** and **b** represents the mean value ± SD of three independent experiments. *, *p* < 0.05, **, *p* < 0.01, ****, *p* < 0.0001. **c** Confocal microscopy images of *S. aureus* SH1000-infected A549 cells showing *S. aureus* in green (middle panel) and CTB-Alexa Fluor 594-bound to GM1 in red (right panel). A merged image of *S. aureus* and CTB-GM1 is shown in the left panel. The scale bar represents 5 μm

Lipid rafts are involved in clathrin-independent endocytic pathways, which are exploited by numerous pathogens for entry into host cells [19–25]. Pathogens internalized via lipid rafts reside in an intracellular compartment that generally do not fuse with traditional lysosomes [26]. Therefore, we investigated the possibility that *S. aureus* could use lipid rafts to enter the human lung epithelial cells. To this end, we determined the effect of lipid rafts disruption using the cholesterol depleting agent β-methylcyclodextrin (MβCD) [48] on bacterial internalization. The results show that *S. aureus* internalization was strongly reduced after treatment with MβCD, indicating that lipid rafts play a crucial role in *S. aureus* entry into A549 (Fig. 3a) or into CI-huBroBECs cells (Supplementary Fig. S5). Furthermore, we observed a reduction in bacterial internalization within A549 cells across different *S. aureus* clinical isolates after MβCD treatment (Fig. 3b). This indicates that the involvement of lipid rafts is not strain-specific but represents a general mechanism used by *S. aureus* strains to invade human lung epithelial cells. To further support the use of lipid rafts by *S. aureus* as an entry mechanism, we examined whether *S. aureus* colocalized with the lipid raft marker ganglioside GM1 in A549 cells. Colocalization of *S. aureus* with GM1 was confirmed using cholera toxin B subunit (CTB), which binds specifically to GM1 [49, 50]. In *S. aureus*-infected A549 cells, CTB-labeled GM1 was recruited to the site where *S. aureus* was attached to A549 cells (Fig. 3c, Supplementary Fig. S6). Collectively, these results support the involvement of lipid rafts in the entry mechanism of *S. aureus* into human lung epithelial cells.

### Caveolin-1 mediates the internalization of *S. aureus* into human lung epithelial cells

**Fig. 4:**
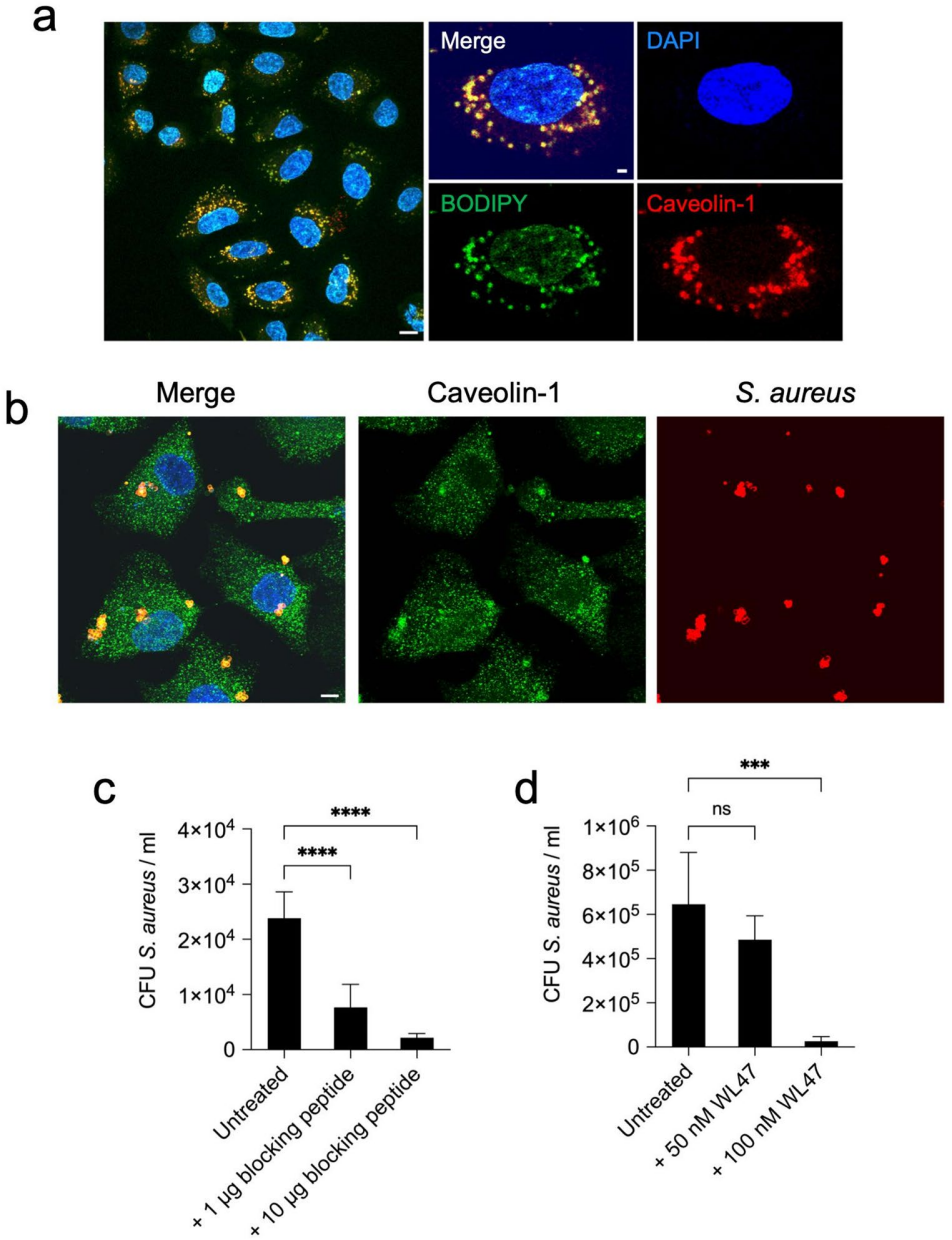
Caveolin-1 mediates *S. aureus* internalization into A549 epithelial cells. **a** Confocal microscopy images of A549 cells stained with BODIPY 493/503 (green) and caveolin-1 (red). DNA in the nucleus is stained in blue. Scale bar represents 5 μm. **b** Confocal microscopy images of A549 cells infected with *S. aureus* SH1000 for 3 h and stained with anti-caveolin-1 and anti-*S. aureus* antibodies. *S. aureus* appears in red (right panel), caveolin-1 in green (middle panel). *S. aureus* colocalizing with caveolin-1 appears in yellow in the merged image (left panel). DNA in the nucleus is stained in blue. Scale bar represents 5 μm. Quantification of viable intracellular *S. aureus* strain SH1000 at 2 h after internalization within A549 cells treated with either 1 µg or10 µg of caveolin-1 blocking peptide (**c**) or with 50 nM or 100 nM of the high-affinity caveolin-1 disruptor WL47 (**d**). Untreated cells are used as controls. Each bar in **c** and **d** represents the mean value ± SD of three independent experiments. ***, *p* < 0.001, ****, *p* < 0.0001

Next, we investigated the specific components associated with lipid rafts that may be used by *S. aureus* to invade human lung epithelial cells. Proteins such as caveolin-1 are enriched in lipid rafts [51], and Hoffmann et al. [27] have reported that caveolin-1 is recruited to the sites of *S. aureus* attachment in fibroblasts. Therefore, we investigated whether caveolin-1 was also recruited to the site of *S. aureus* attachment in A549 lung epithelial cells. Since the expression levels of caveolin-1 can vary between different cell types [52], we first determined the expression levels of caveolin-1 in A549 cells. We found that A549 cells expressed high levels of caveolin-1, which colocalized with lipids in the cell membrane (Fig. 4a). Confocal microscopy examination of infected A549 cells revealed colocalization of *S. aureus* with caveolin-1, suggesting a potential role for caveolin-1 in the internalization process (Fig. 4b). The relevance of caveolin-1 for bacterial internalization was further supported by the dose-dependent reduction in the number of intracellular *S. aureus* observed after disruption of caveolin-1 functionality with either a blocking peptide that inhibits the interaction of caveolin-1 with potential ligands (Fig. 4c) or with the caveolin-1 oligomer disruptor WL47 (Fig. 4d).

### Hla is crucial for the internalization of *S. aureus* into human lung epithelial cells

**Fig. 5:**
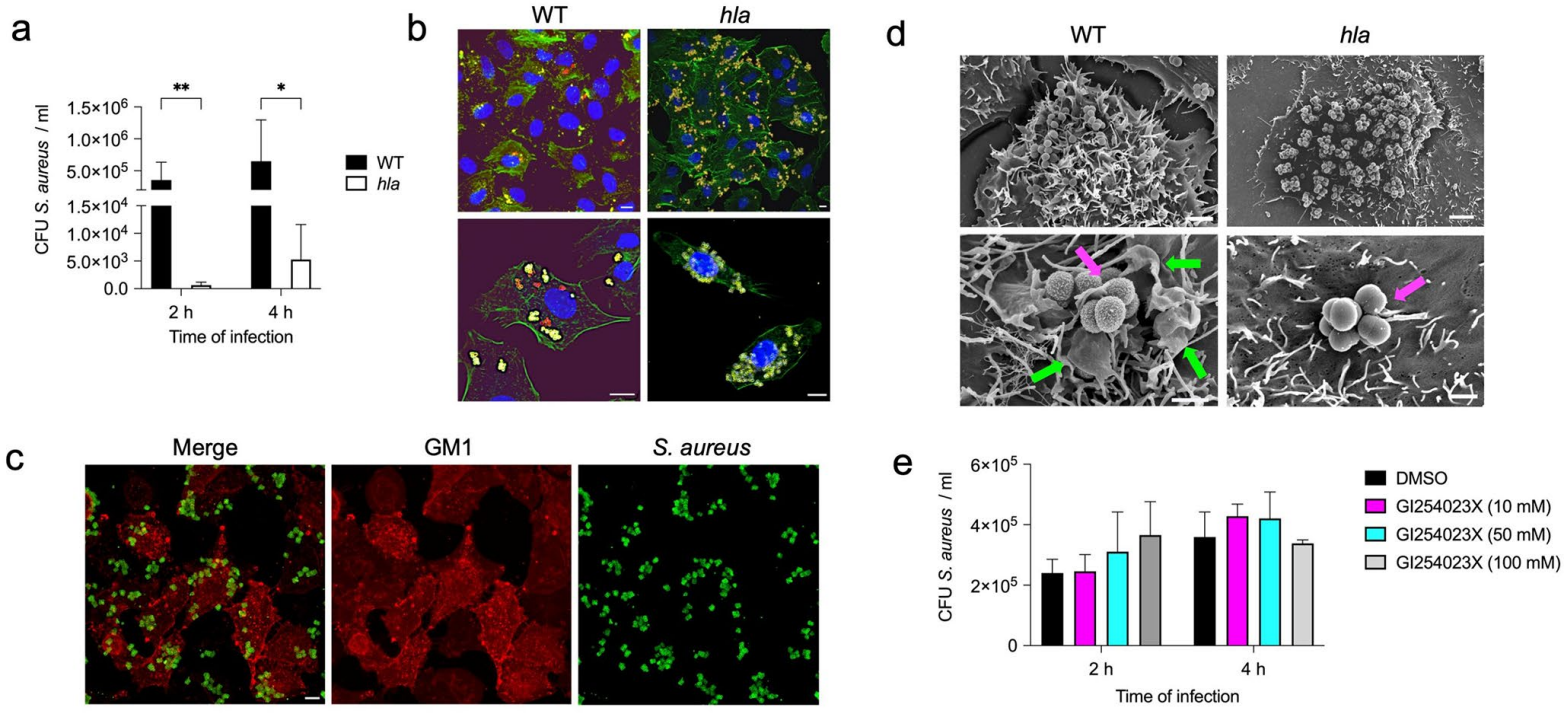
Hla is required by *S. aureus* for internalization into human lung epithelial cells. **a** Quantification of intracellular viable *S. aureus* wild-type (WT) (black bars) and the corresponding *S. aureus* mutant strain deficient in the expression of Hla (*hla*) (white bars) within A549 cells at 2 h and 4 h of infection. Each bar represents the mean value ± SD of three independent experiments. *, *p* < 0.05, **, *p* < 0.01. **b** Immunofluorescence microscopy images showing A549 cells infected for 4 h with either *S. aureus* WT (left panels) or with the corresponding Hla-deficient *S. aureus* strain (right panels). Intracellular bacteria appear red, extracellular bacteria appear yellow, cell actin cytoskeleton appears green and DNA in the nucleus is stained in blue. **c** Immunofluorescence microscopy images showing A549 cells infected with *S. aureus hla* for 4 h and stained with CTB-Alexa Fluor 594 to identify GM1. *S. aureus* appears green (right panel) and CTB-GM1 appears red (middle panel), the merged image is shown in the left panel. Scale bars are 10 μm. **d** Scanning electron microscopy images showing A549 cells infected with either *S. aureus* WT (left panels) or with the corresponding Hla-deficient *S. aureus* strain (right panels) for 4 h. Magenta arrows indicate *S. aureus* bacteria and green arrow indicates membrane ruffles. Scale bars are 2 μm for the upper left panel, 5 μm for the upper right panel and 1 μm for the lower panels. **e** Quantification of intracellular viable *S. aureus* strain SH1000 in A549 cells at 2 h and 4 h of infection treated with either different concentrations of the caveolin-1 inhibitor GI254023X or vehicle DMSO. Each bar represents the mean value ± SD of three independent experiments

The above results indicated that the interaction of *S. aureus* with caveolin-1 in A549 cells was critical for bacterial internalization. Therefore, we next investigated the potential virulence determinant of *S. aureus* that could mediate this interaction. In this regard, Hla is the only virulence factor of *S. aureus* that has been shown to directly interact with caveolin-1 [53–56]. Hla is a pore-forming protein produced by almost all *S. aureus* strains and plays an important role in the pathogenesis of *S. aureus* infections [29]. Hla is secreted as a monomer by *S. aureus* and forms heptameric pores in the membranes of target host cells [29]. Hla has also been reported to induce extensive clustering of caveolin-1 on the surface of A431 epithelial cells [54]. Based on these observations, we investigated the role of Hla in *S. aureus* internalization into human lung epithelial cells. We found that a mutant *S. aureus* strain deficient in the expression of Hla (*S. aureus hla*) was significantly impaired in its ability to internalize into human lung epithelial cells, as evidenced by the significantly lower numbers of intracellular bacteria detected in infected A549 (Fig. 5a) or in infected CI-huBroBEC cells (Supplementary Fig. S7a) cells at 2 h and 4 h postinfection. Confocal microscopy of infected A549 cells (Fig. 5b and Supplementary Fig. S8) and of infected CI-huBroBEC cells (Supplementary Fig. S7b) showed that, in contrast to *S. aureus* wild-type (WT), *S. aureus hla* was unable to internalize into human epithelial cells and remained attached to the cell surface.

Confocal microscopy examination of A549 cells infected with *S. aureus hla* also revealed a lack of colocalization of *S. aureus* with CTB-GM1, suggesting that *S. aureus hla* was unable to induce lipid raft formation at the adhesion site in A549 cells (Fig. 5c). Further examination of A549 cells infected with *S. aureus* WT or *hla* by scanning electron microscopy confirmed that *S. aureus hla* had a comparable capacity to adhere to the surface of A549 cells (Fig. 5d, upper right panel) as *S. aureus* WT (Fig. 5d, upper left panel). However, in contrast to *S. aureus* WT (Fig. 5d, lower left panel), *S. aureus hla* was unable to induce membrane ruffling around the attached bacteria (Fig. 5d, lower right panel). Taken together, these data demonstrate that Hla is required for *S. aureus* internalization in human lung epithelial cells.

In addition to caveolin-1, ADAM10 has been reported to be a receptor for Hla in lipid rafts [57]. To determine the relevance of ADAM10 in the internalization process, we examined the effect of treating A549 epithelial cells with different concentrations of the ADAM10-specific inhibitor GI254023X [58] on bacterial internalization. The results in Fig. 5e show that inhibition of ADAM10 did not affect the capacity of *S. aureus* to internalize into A549 cells. These results suggest that ADAM10 is not involved in the interactions of *S. aureus* with lipid rafts in human lung epithelial cells.

### Hla is detected in the membrane ruffles of lung epithelial cells in close proximity to attached *S. aureus*

**Fig. 6:**
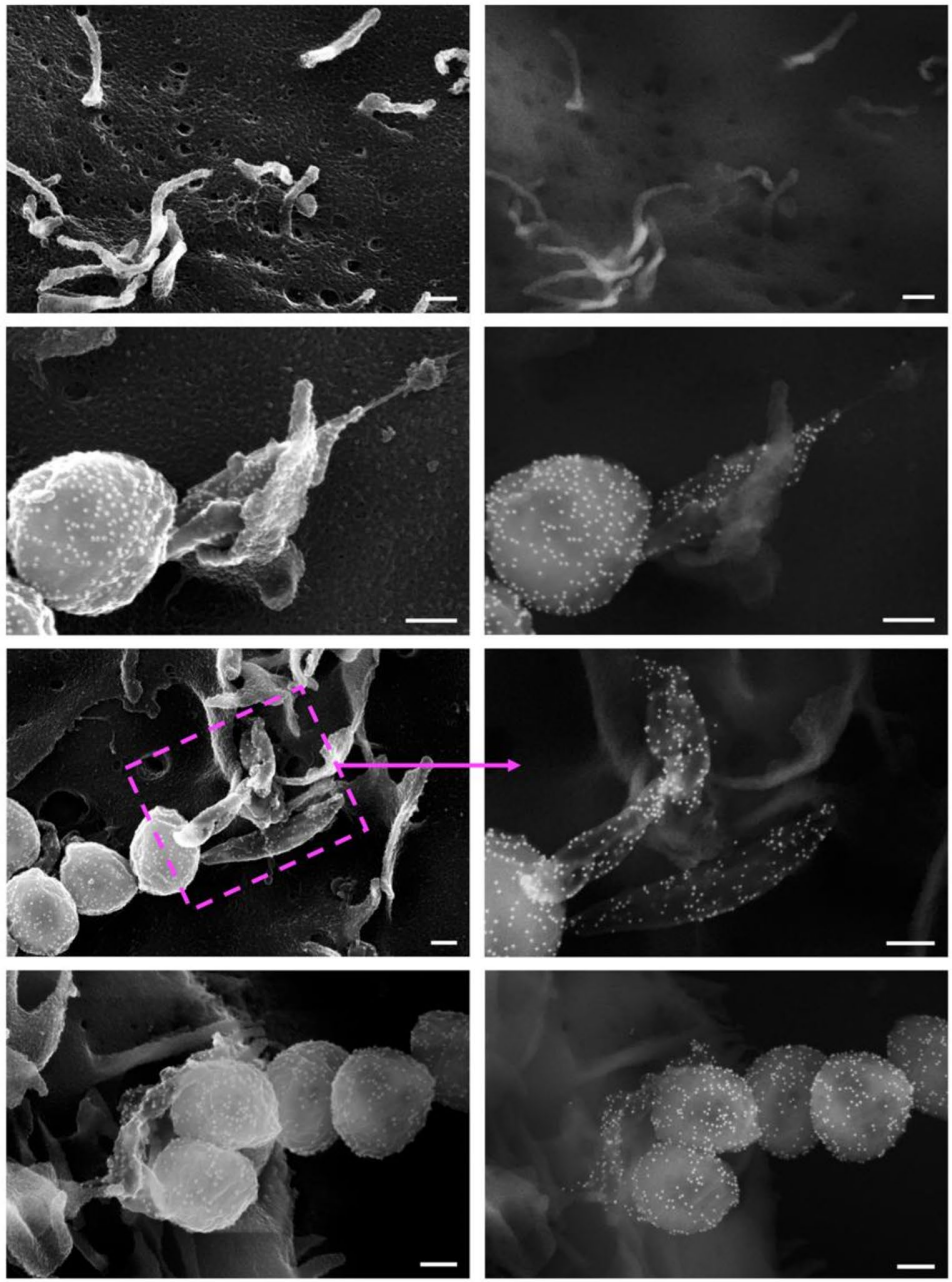
Hla secreted by *S. aureus* is bound to bacteria-associated membrane ruffles in A549 epithelial cells. FESEM images showing uninfected A549 cells (first row panels) and A549 cells infected with *S. aureus* SH1000 for 4 h (other panels) and immunogold labeled with anti-Hla primary antibodies and secondary antibodies tagged with 15 nm gold particles. The left panels were photographed using the InLens detector and the right panels were photographed using the HE-SE2 detector to highlight the gold labeling. The inset shows a magnified view of the area inside the magenta box. Scale bars represent 0.2 μm

*S. aureus* produces Hla as a soluble monomer that is secreted into the extracellular environment. Hla is secreted by *S. aureus* via the general secretory pathway and is cleaved at the bacterial cell surface by type I signal peptidase [59]. Therefore, we hypothesized that secreted Hla might interact with caveolin-1 near the bacterial binding site in the membrane of lung epithelial cells, leading to membrane ruffling and bacterial internalization. To test this hypothesis, Hla was labeled in *S. aureus-*infected A549 cells using anti-Hla antibodies conjugated to gold particles and examined by scanning electron microscopy. The images shown in Fig. 6 confirm the presence of Hla predominantly in the membrane ruffles that are in close proximity to *S. aureus* in A549 cells.

**Fig. 7:**
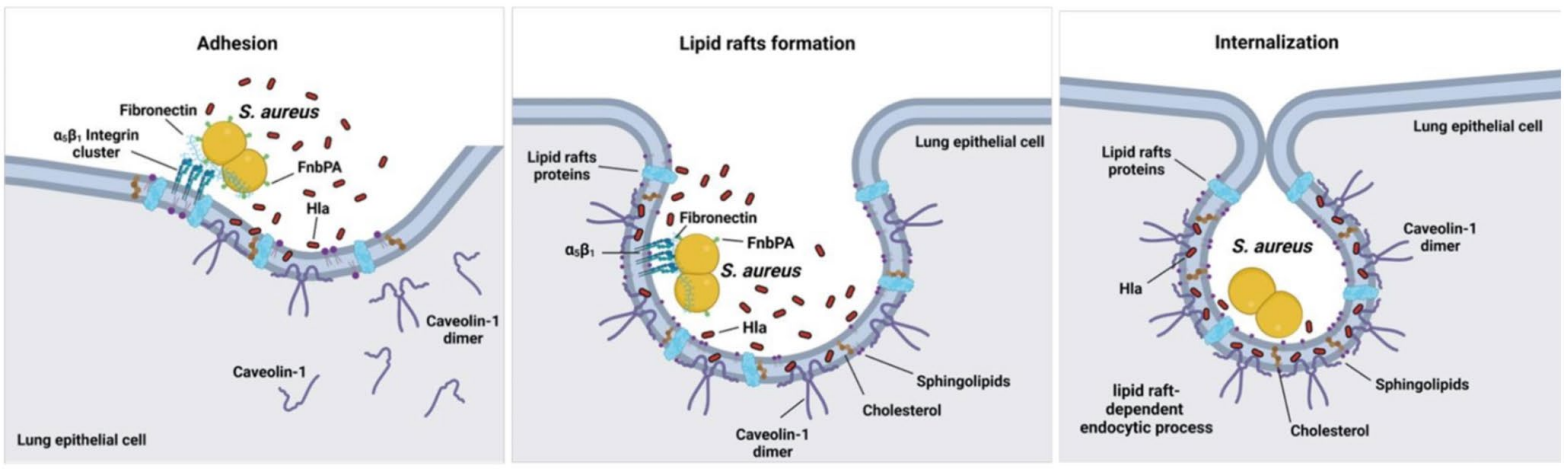
Schematic representation of the proposed model for *S. aureus* internalization into human lung epithelial cells via Hla interaction with caveolin-1 on lipid rafts. The first step is the adhesion of *S. aureus* to lung epithelial cells, which involves the α5β1 integrin expressed on the epithelial cell and is mediated by bacterial fibronectin-binding protein A (FnbPA) via fibronectin as a connecting molecule (left panel). Hla released by the attached *S. aureus* interacts with caveolin-1 in the epithelial cell membrane, leading to membrane destabilization and formation of sphingolipid- and cholesterol-rich lipid rafts surrounding the bacteria (middle panel). The lipid rafts then collapse onto the plasma membrane, where they fuse and then split to form and intracellular vacuole containing *S. aureus* (right panel). The scheme was created using BioRender.com

A schematic representation of the interactions between Hla, lipid rafts, and caveolin-1 that facilitate *S. aureus* internalization into lung epithelial cells is shown in Fig. 7.

## Discussion

In this study, we investigated the molecular mechanisms underlying *S. aureus* internalization into human lung epithelial cells. We provide evidence that *S. aureus* internalization within these cells is facilitated by lipid raft-mediated endocytosis. Accordingly, treatment of A549 or CI-huBroBECs cells with the lipid raft disrupting drug MβCD significantly impaired *S. aureus* internalization. Attachment of *S. aureus* to α5β1 integrin on the surface of lung epithelial cells via fibronectin bound to bacterial FnBPs was a prerequisite for bacterial internalization. However, a major finding of this study was that the interaction between bacterial Hla and caveolin-1 on the host cell appeared to be essential for the internalization process. This was supported by the observation that a mutant *S. aureus* strain deficient in the expression of Hla was significantly impaired in bacterial internalization despite efficient attachment to the epithelial cell surface. Similarly, inhibition of caveolin-1 functionality on lung epithelial cells resulted in a significant reduction in *S. aureus* internalization.

Hla is a beta-barrel pore-forming toxin that assembles into heptameric pores in the membrane of target cells [29]. This toxin is one of the major virulence factors involved in the pathogenesis of *S. aureus* lung infections [29, 60]. The binding of Hla to target cells typically involves receptor-dependent and receptor-independent mechanisms [61]. At high concentrations (> 1 µM), Hla binds to most eukaryotic cells in a receptor-independent manner. This type of binding may occur through interactions with specific membrane lipids such as phosphocholine head groups [28]. At low concentrations (1–2 nM), Hla binds to target cells via specific receptors such as ADAM10 [57] and caveolin-1 [53–56, 62]. In this study, we found that while caveolin-1 was essential, ADAM10 was dispensable for *S. aureus* internalization into lung epithelial cells. There is considerable evidence for a direct interaction between *S. aureus* Hla and caveolin-1 in the eukaryotic cell membrane [53, 54, 56]. For example, the addition of purified caveolin-1 has been shown to inhibit Hla-induced hemolysis of rabbit erythrocytes in a dose-dependent manner [56]. Furthermore, Hla was found to co-precipitate with caveolin-1 in pull-down assays, suggesting a physical association between Hla and caveolin-1 at the molecular level [54]. This was further supported by the colocalization of Hla with caveolin-1 observed in A431 epithelial cells by confocal microscopy [54].

A major remaining question in our study is how extracellular Hla, which is secreted by *S. aureus* and diffusing through the extracellular milieu, can bind to caveolin-1, which is generally believed to be located on the cytoplasmic side of the cell membrane. In this regard, it has been proposed that the aromatic tip of the scaffolding domain of caveolin-1 is positioned within the membrane bilayer where it may be accessible from the extracellular environment [63]. The rest of the protein is located in the cytoplasm, with the N-terminal region functioning as a scaffolding domain that controls lipid raft-dependent endocytosis [64]. Thus, extracellular Hla could potentially interact with the exposed aromatic tip of caveolin-1 at the membrane interface, leading to subsequent internalization events. Support for this statement is provided by the high levels of Hla detected primarily in the lipid rafts surrounding *S. aureus* in human lung epithelial cells (Fig. 5). Interestingly, Hoffmann et al. [27] reported that, in fibroblasts, caveolin-1 acts as a lock that stabilizes the plasma membrane in the lipid rafts [27]. This implies that specific signaling events may be required to induce conformational changes or modifications in caveolin-1 that allow it to destabilize the cell membrane and initiate the lipid raft-dependent endocytic process. Based on the results of our study, we propose that the interaction between Hla and caveolin-1 leads to cell membrane destabilization in lung epithelial cells to allow *S. aureus* endocytosis via lipid rafts.

The use of lipid rafts and caveolin-1 to enter host cells is not unique to *S. aureus* but is rather a common strategy used by various pathogens from different taxa to gain access to their host cells [19, 20, 22, 23, 25, 26]. This underscores the evolutionary advantage of this cell invasion strategy for pathogen survival. Pathogen entry via lipid rafts prevents fusion with lysosomes and subsequent degradation by the host antimicrobial mechanisms [24]. Indeed, we found that *S. aureus* was not only able to survive in the intracellular compartment of human lung epithelial cells but also remained viable and even proliferated. In addition to lung epithelial cells, *S. aureus* is able to invade and survive intracellularly in many other cell types [65]. This ability allows *S. aureus* to hide from the immune cells and persist in the host. In addition, many antibiotics are poorly penetrating into eukaryotic cells and do not reach the concentration necessary to eliminate intracellular *S. aureus*, leading to treatment failure and even promoting antibiotic tolerance [66]. Consequently, elimination of the intracellular *S. aureus* reservoir is key to treatment success. An alternative to killing intracellular *S. aureus* would be to interfere with its ability to internalize into host cells, for example by inactivating the bacterial virulence determinants involved in the internalization process. Since the pathway used by *S. aureus* for internalization may vary depending on the infected cell type, understanding the pathways and virulence factors involved in its invasion of different host cells is critical for developing effective strategies to target the intracellular bacterial reservoir. In the specific case of human lung epithelial cells, this study shows that inhibiting the interaction between bacterial Hla and host cell caveolin-1 significantly reduces the ability of *S. aureus* to internalize within these cells. In this regard, Hla inhibitors such as anti-Hla antibodies in combination with antimicrobial treatment, may represent a more effective strategy for successful treatment of *S. aureus* respiratory infections.

## Electronic supplementary material

Below is the link to the electronic supplementary material.

Supplementary Material 1

## Data Availability

All data generated or analyzed during this study are included in this article and its supplementary information file. Additionally, data are available from the corresponding author upon request.
