# Supplementary material for: Alpha-hemolysin promotes internalization of Staphylococcus aureus into human lung epithelial cells via caveolin-1- and cholesterol-rich lipid rafts
Source: Cell Mol Life Sci. 2024 Oct 16;81(1):435. doi: 10.1007/s00018-024-05472-0 (PMC11488825; doi:10.1007/s00018-024-05472-0)
Supplement: Supplementary file 1 — Supplementary Material 1 [file 18_2024_5472_MOESM1_ESM.pdf]

Supplementary Figure S1

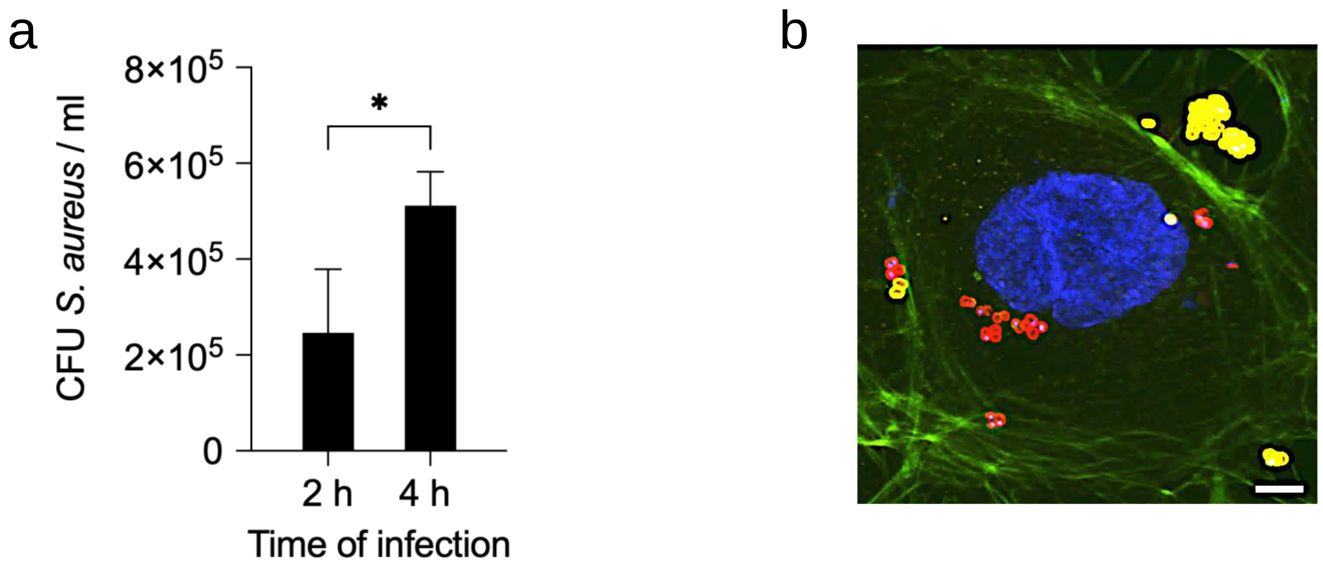

Fig. S1 Internalization of *S. aureus* into human CI-huBroBEC bronchial basal epithelial cells. **a** Quantification of viable intracellular *S. aureus* strain SH1000 in CI-huBroBEC cells at 2h and 4 h of infection. Each bar represents the mean value ± SD of three independent experiments. *, $p < 0.05$. **b** Double immunofluorescence staining images of *S. aureus* SH100-infected CI-huBroBEC cells at 4 h of infection showing the intracellular bacteria in red, extracellular bacteria in yellow, actin in green and DNA in the nucleus is stained in blue. Scale bar represents 5 µm.

Supplementary Figure S2

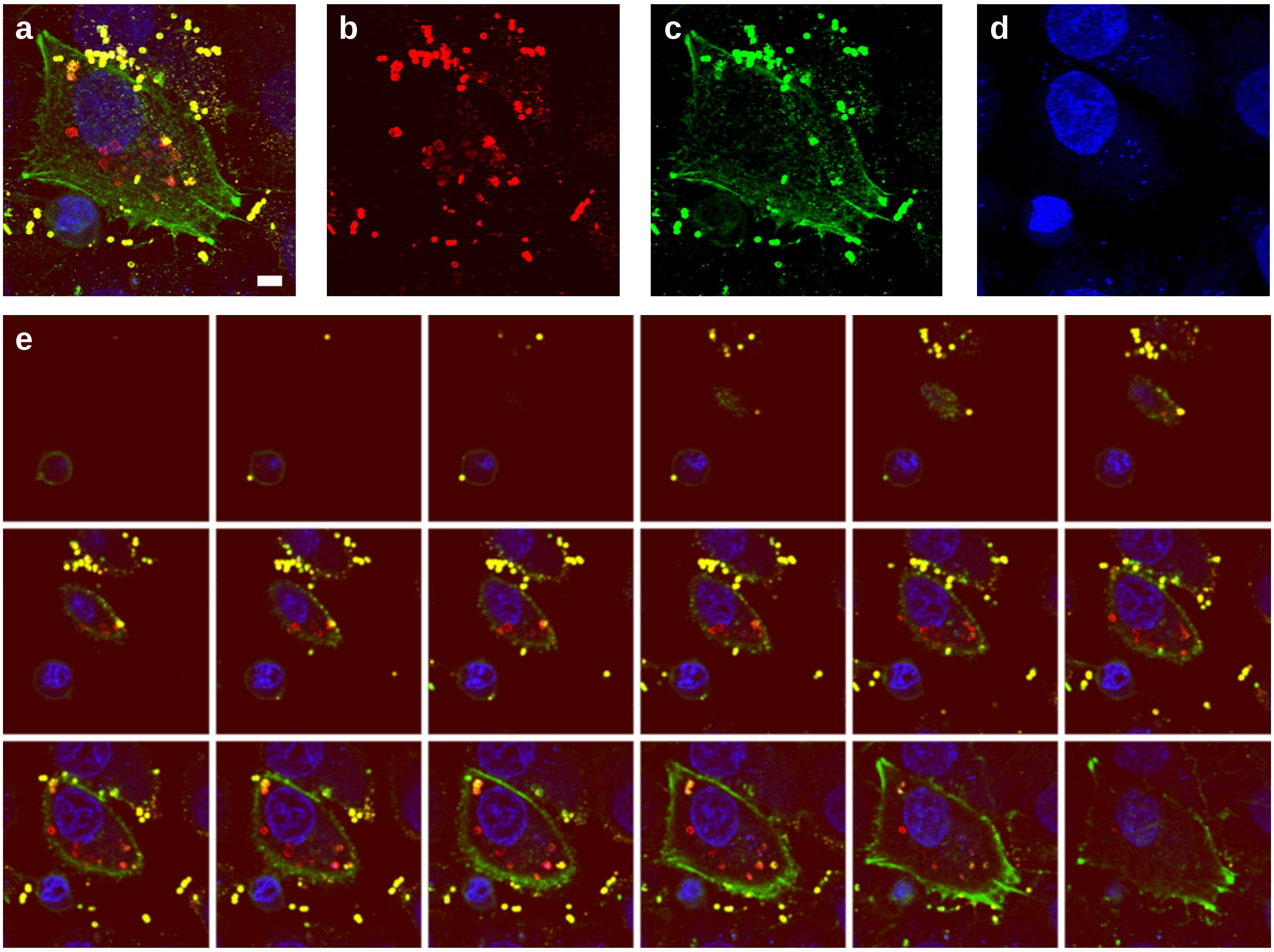

Fig. S2 Adhesion to and internalization of *S. aureus* into A549 epithelial cells. **a** Composite maximun projection image (Z-Volume: 8.56 µm) of 18 optical sections (each 0.5 µm thick) showing *S. aureus* internalized within A549 cells at 4 h of infection. Intracellular bacteria are stained red, extracellular bacteria are stained yellow, actin is stained green and DNA in the nucleus is stained in blue. Scale bar represents 5 µm. Maximun projection images of red channel (Alexa Fluor 568) are shown in **b**, from green channel (Alexa Fluor 488) in shown in **c** and from DAPI in **d**. **e** Confocal z-stack images from top to bottom, each step for z-stack images is 0.5 µm.

Supplementary Figure S3

S. aureus WT *S. aureus FnBPA*

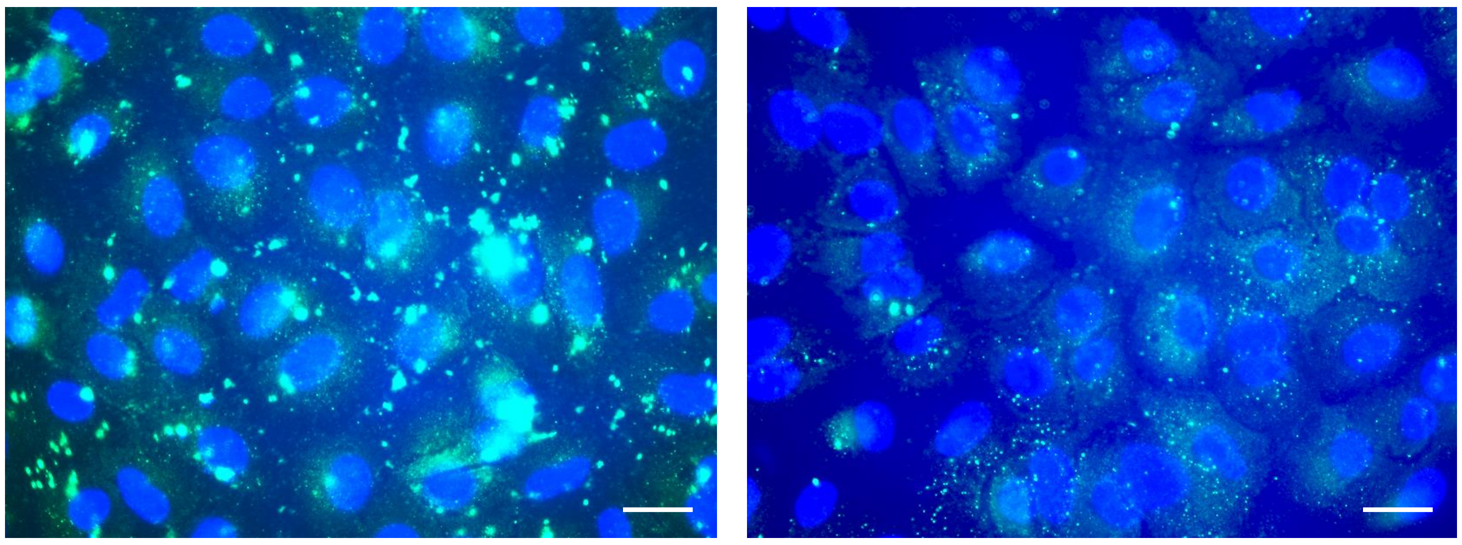

Fig. S3 Adhesion of carboxyfluorescein-labeled *S. aureus* wild-type (left) and *S. aureus* FnBPA-deficient mutant (right) to A549 epithelial cells. *S. aureus* appears green and DNA in the nucleus of A549 cells is stained in blue. Bars represent 10 μM.

Supplementary Figure S4

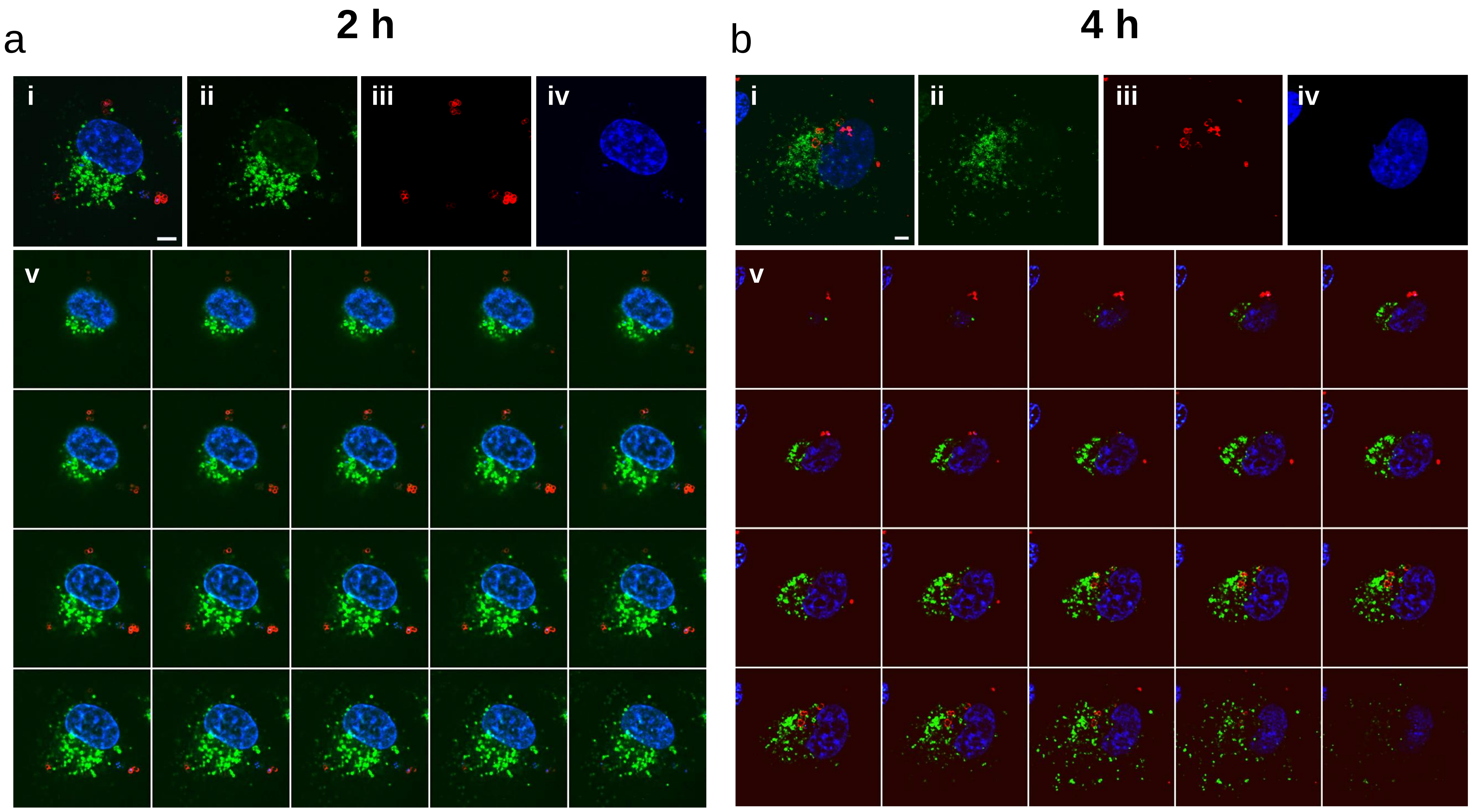

Fig. S4 Intracellular *S. aureus* does not colocalize with LAMP-1 within human lung epithelial cells. Confocal microscopy images of *S. aureus* SH1000-infected A549 cells for either 2 h (**a**) or 4 h (**b**) showing LAMP-1 in green and *S. aureus* in red and DNA in blue. A composite maximum projection image (z-Volume: 5.97 µm) of 35 optical sections (each 0.17 µm thick) is shown in (**i**). Maximun projection images of Alexa 488 (LAMP-1) channel are shown in **(ii)**, of Alexa 568 (*S. aureus* SH1000) in (**iii**) and of DAPI (DNA) in (**iv**). Scale bars are 5 µm. (**v**) Confocal z-stack images from top to bottom of 20 optical sections (from slice 15 to 35), each step for z-stack images is 0.17 µm.

Supplementary Figure S5

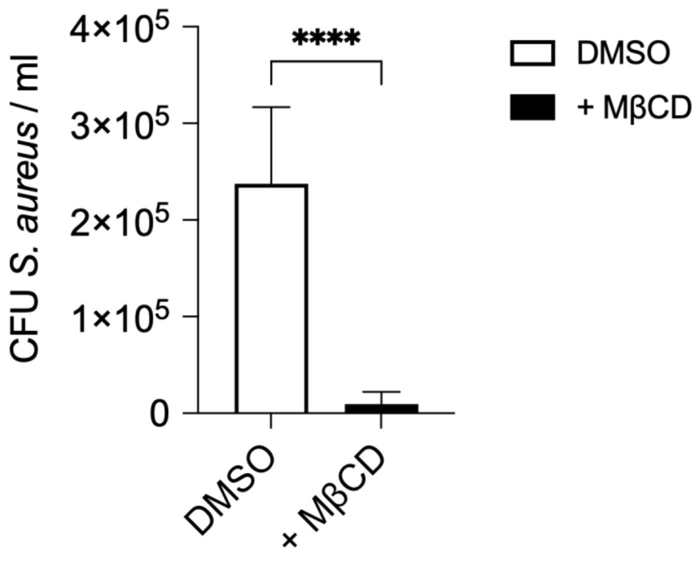

Fig. S5 *S. aureus* enters human CI-huBroBEC cells via lipid raft-mediated endocytosis. Quantification of viable bacteria within CI-huBroBEC cells treated with either 10 mM MβCD (black bars) or with DMSO vehicle control (white bars) after 2 h of infection with *S. aureus* strain SH1000. Each bar represents the mean value ± SD of three independent experiments. ****, $p < 0.0001$.

Supplementary Figure S6

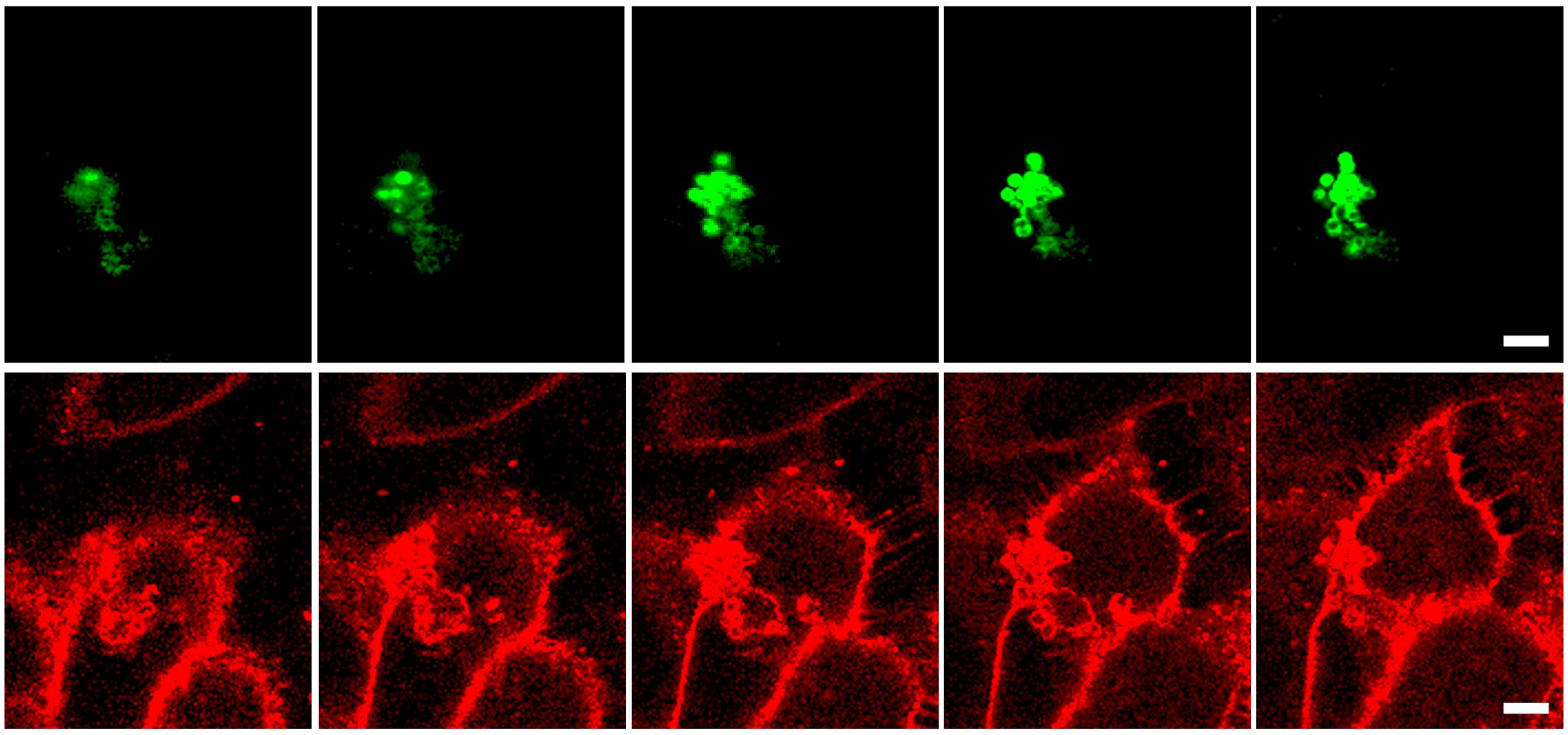

Fig. S6 Laser scanning confocal microscope images of six consecutive optical sections of 0.59 µm thickness each (sections 9 to 13) from the maximum intensity projections shown in the Fig. 2e. *S. aureus* attached to the surface of A549 cells appears green (top panels) and CTB-GM1 appears red (bottom panels). Scale bar represents 5 µm.

Supplementary Figure S7

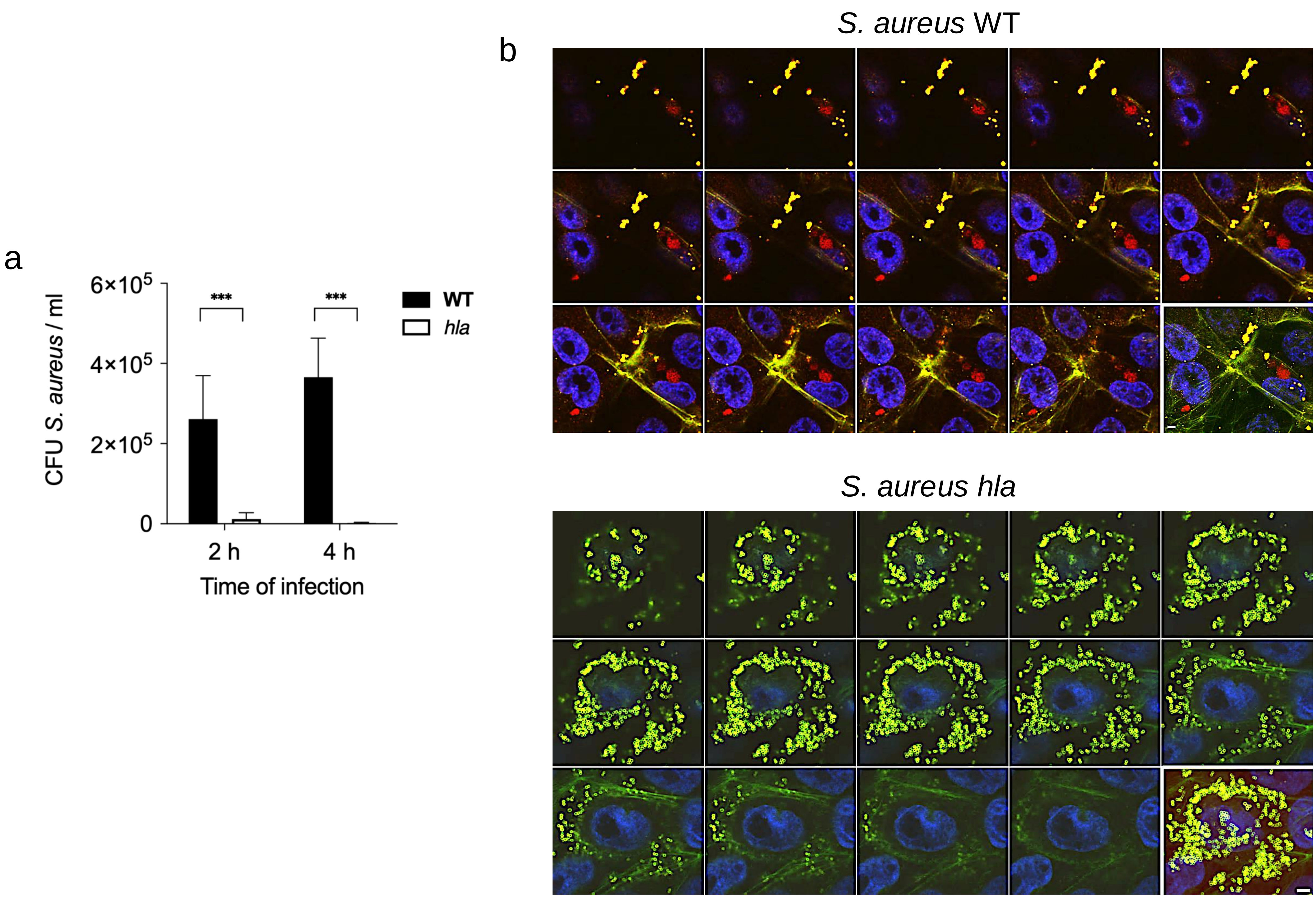

Fig. S7 Hla is required for *S. aureus* internalization within CI-huBroBEC cells. **a** Quantification of intracellular viable *S. aureus* wild-type (WT) (black bars) and the corresponding *S. aureus* mutant strain deficient in the expression of Hla (*hla*) (white bars) within CI-huBroBEC cells at 2 h and 4 h of infection. Each bar represents the mean value ± SD of three independent experiments. ***, $p < 0.001$. **b** Confocal immunofluorescence images showing CI-huBroBEC cells infected with either *S. aureus* strain SH1000 WT (upper panels) or with the Hla-deficient strain (lower panels) for 4 h. Intracellular bacteria appear red, extracellular bacteria yellow, cell actin cytoskeleton appears green and DNA in the nucleus is stained in blue. Bars represent 5 µM.

Supplementary Figure S8

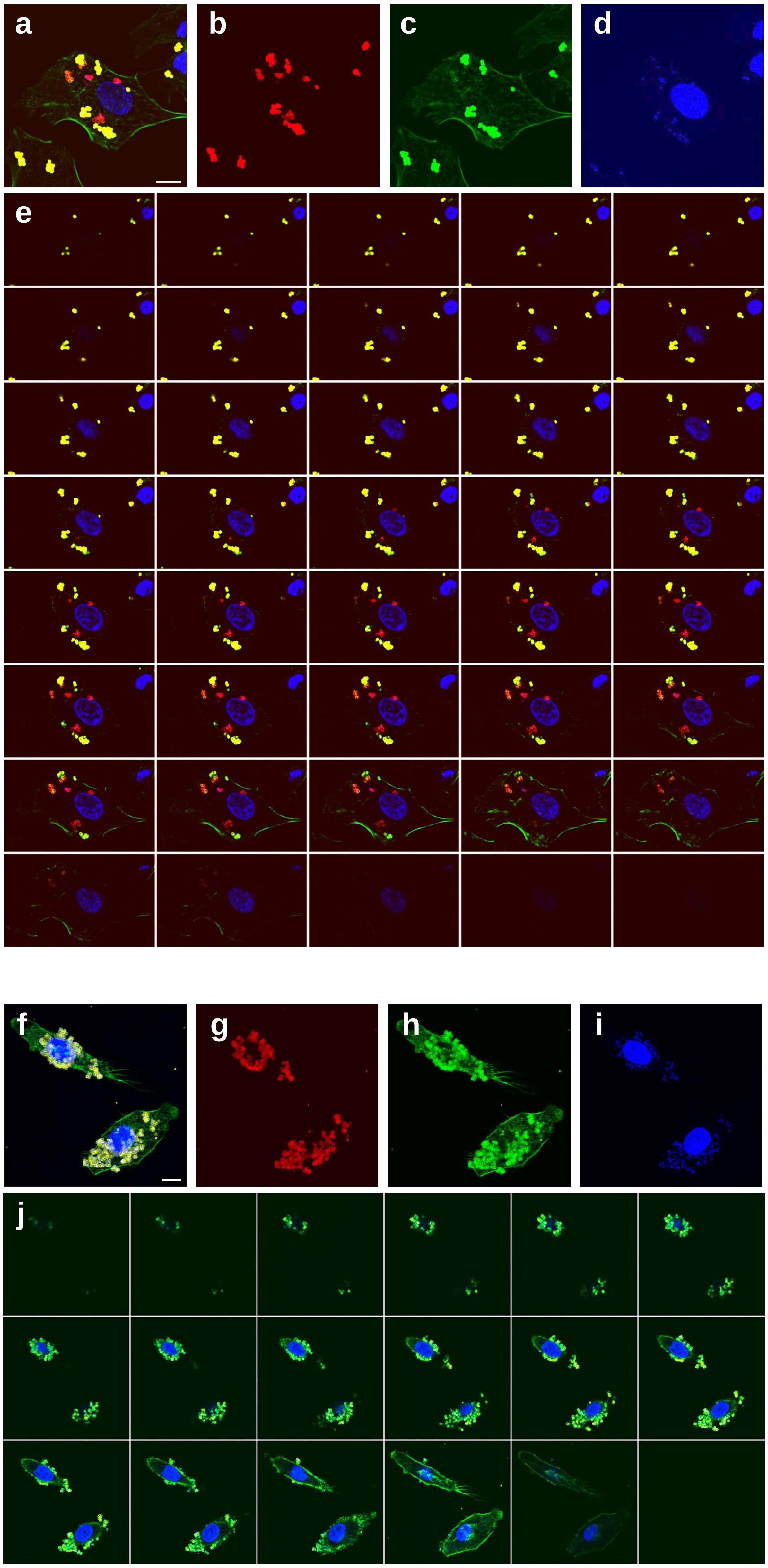

Fig. S8 Hla is required for *S. aureus* internalization within human lung epithelial cells. **a** Composite maximun projection image (z-Volume: 9.90 µm) of 60 optical sections (0.17 µm thick each) showing A549 cells infected with *S. aureus* WT at 4 h of infection. Intracellular bacteria are stained in red, extracellular bacteria are stained yellow, actin is stained green and DNA in the nucleus is stained in blue. Maximun projection images of the red channel (Alexa Fluor 568) are shown in **b**, of the green channel (Alexa Fluor 488) are shown in **c** and of DAPI in **d**. **e** Confocal z-stack images from top to bottom of 40 optical sections (from 10 to 50), each step for Z-stack images is 0.17 µm. **f** Composite maximal projection image (z-Volume: 10.742 µm) from 17 optical sections (each 0.67 µm thick) showing showing A549 cells infected with *S. aureus hla* at 4 h of infection. Maximun projection images of each channel are shown in **g**, **h** and **i**. **j** Confocal Z-stack images from top to bottom of 17 optical sections, each step for z-stack images is 0.67 µm. Scale bars are 10 µm in **a** and **f**.
